# Supplementary material for: Resolving the full spectrum of human genome variation using Linked-Reads
Source: Genome Res. 2019 Apr;29(4):635–45. doi: 10.1101/gr.234443.118 (PMC6442396; doi:10.1101/gr.234443.118)
Supplement: Supplemental Material [file supp_gr.234443.118_Supplemental_longranger-2.2.zip › longranger-2.2.tar.bz2!/lr2.2-public/lib/go/src/code.google.com/vitess/vitess.io/_includes/navigation-sliding.html]

{% for link in site.data.navigation %}- {% unless link.url contains 'http' %}
  {% assign domain = site.url %}
  {% endunless %}
  {% if link.image %}{% endif %}
  {{ link.title }}
  {% if link.excerpt %}

  {{ link.excerpt }}

  {% endif %}
{% endfor %}
